# A Case Series of Isolated Primary Hydatid Cyst of the Spleen

**DOI:** 10.7759/cureus.62252

**Published:** 2024-06-12

**Authors:** Tarun C Mulpuri, Yadavalli RD Rajan, Joshua Sajja, Sai Raja Sekhar Gogineni, Sri Sai Anvita Veerapaneni

**Affiliations:** 1 General Surgery, Siddhartha Medical College, Vijayawada, IND; 2 General Medicine, Siddhartha Medical College, Vijayawada, IND

**Keywords:** splenectomy, echinococcus granulosis, primary splenic hydatid, cyst hydatid, spleen

## Abstract

Hydatid disease is endemic mainly in Asia and other sheep-raising areas. In India, hydatid disease has been a very common disease because of its close association with livestock rearing. Hydatid disease is a parasitic infestation caused by Echinococcus granulosus. The usual location of infestation is in the liver sinusoids, lungs, and spleen. Hydatid disease in humans is rare, and a hydatid cyst of the spleen without involving the liver is very rare. The rarity of splenic hydatid disease may pose a diagnostic challenge for clinicians, especially in non-endemic areas. The diagnosis of hydatid disease is based on the epidemiological background of patients, clinical grounds, or noninvasive screening procedures. With this background, we aimed to study the presenting symptomatology and various clinical manifestations of isolated spleen hydatid disease and analyze the morbidity and mortality of hydatid disease. Different surgical modalities and their complications were studied. Three patients operated on for splenic hydatid at our institute were studied retrospectively.

## Introduction

Hydatid disease exhibits endemicity mostly in Mediterranean countries, the Middle East, Baltic regions, South America, India, northern China, and other geographical locations where sheep-raising is prevalent. Hydatid illness is prevalent in various regions of India, with a particularly high incidence observed in Andhra Pradesh and Tamil Nadu [1]. Hydatid illness, a parasitic infection, occurs in humans as an unintended host. The liver is the primary location of infection, with subsequent occurrences observed in the lungs, kidneys, bones, and the brain. Infrequently, other anatomical areas, such as the heart, spleen, pancreas, and muscles, exhibit manifestations [2]. The spleen is the third most common organ involved after the liver and the lungs. Patients present mostly asymptomatic, while some present with dull pain in the left side of the abdomen. The diagnostic challenge for doctors may be heightened because of the infrequency of splenic hydatid disease, particularly in regions where it is not endemic. The diagnosis of hydatid disease relies on several factors, including the epidemiological history of individuals, clinical manifestations, and noninvasive screening measures [3]. A retrospective study was conducted on three patients who underwent surgical treatment for splenic hydatid at our institution.

## Case presentation

Case 1

**Figure 1:**
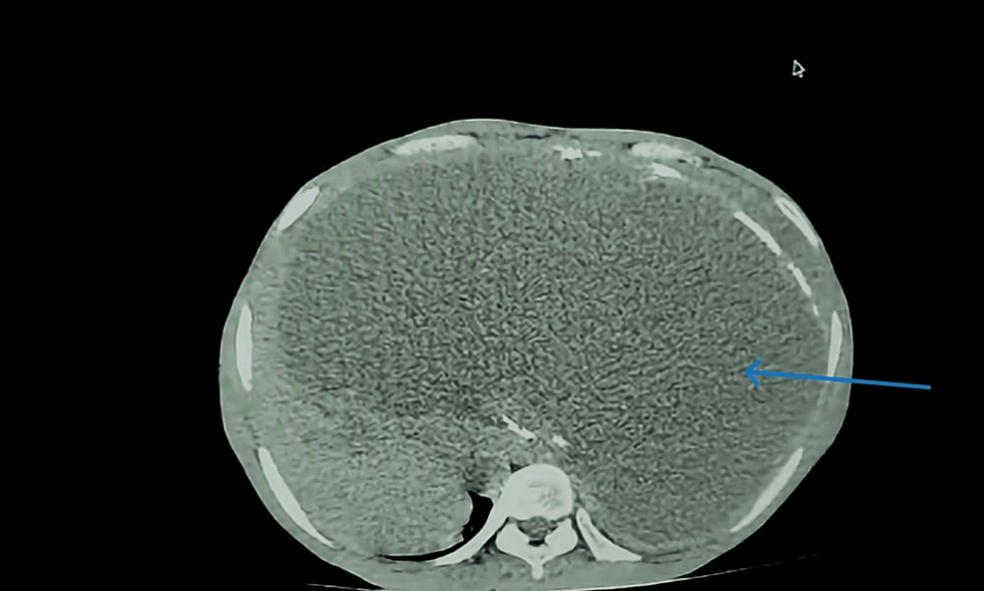
A CT scan of the abdomen showing a large fluid-filled lesion occupying most of the abdomen The blue arrow represents the hydatid cyst

**Figure 2:**
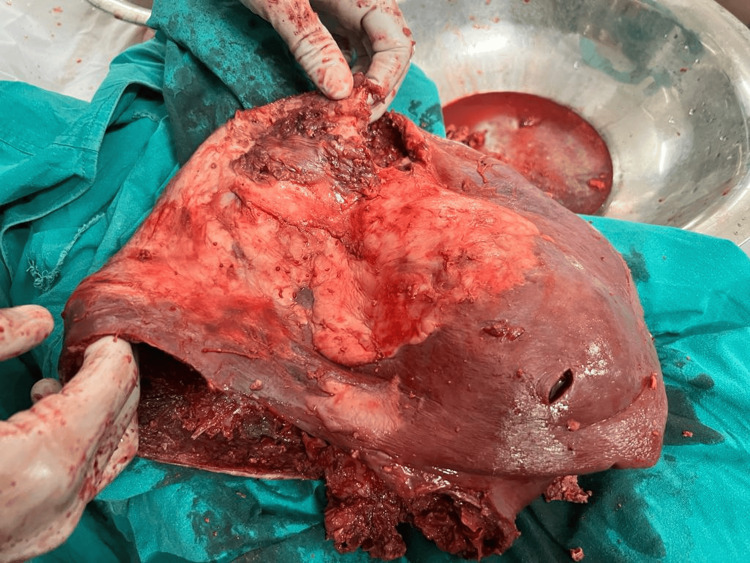
Postoperative specimen showing the spleen along with the capsule

A 45-year-old male cattle handler from Andhra Pradesh presented to the outpatient department with complaints of vomiting, and pain abdomen, and identified mass per abdomen for the past six months. Vomiting aggravated after food intake and subsided in a few hours, pain was a dull dragging type with no radiation. On clinical examination, a mass was observed crossing the midline and covering both hypochondria, epigastrium, umbilical, and left iliac and lumbar region. Laboratory blood tests did not lead to any conclusion. A CT of the abdomen showed a 15 x 28 x 16 cm large well-defined heterogeneous fluid density lesion noted in the abdomen (bilateral hypochondrium, epigastrium, umbilical region‚ left lumbar region, and iliac fossa) (Figure 1). The lesion shows intralesional and peripheral calcifications. Multiple ill-defined irregular soft tissue density areas are noted within the lesion. Solid components of the lesion show minimal heterogeneous enhancement on IV contrast. The lesion is seen indenting the right and left lobes of the liver. The stomach and pancreas were pushed posteriorly and toward the right by the mass lesion. The spleen was pushed inferiorly by the mass lesion. The lesion was causing compression over the left kidney. The lesion shows loss of fat planes with adjacent structures (e.g., liver, stomach, colon, pancreas, spleen, and left kidney). The aorta and inferior vena cava (IVC) are pushed towards the right by the mass lesion. The intrahepatic part of the IVC is compressed by the mass lesion. The intrahepatic part of IVC and bilateral common iliac veins show no contrast filling. Differential outcomes include a large mesenteric cyst and a hydatid cyst. An exploratory laparotomy was performed, and a large cyst arising from the spleen crossing midline and adherent to the left dome of the diaphragm was visualized. Internal calcification and septations were noted in the cyst. A total splenectomy was done (Figure 2), followed by an intra-abdominal wash with 3% NaCl and metronidazole considering the cyst to be a hydatid cyst. The postoperative period was uneventful. The patient was kept on oral albendazole 400 mg twice a day for 28 days. Pneumococcal and influenza vaccines were given postoperatively. The biopsy proved to be a chronic calcified hydatid cyst of the spleen. On follow-up at six-month intervals, ultrasonography showed no recurrence or specific complaints.

Case 2

**Figure 3:**
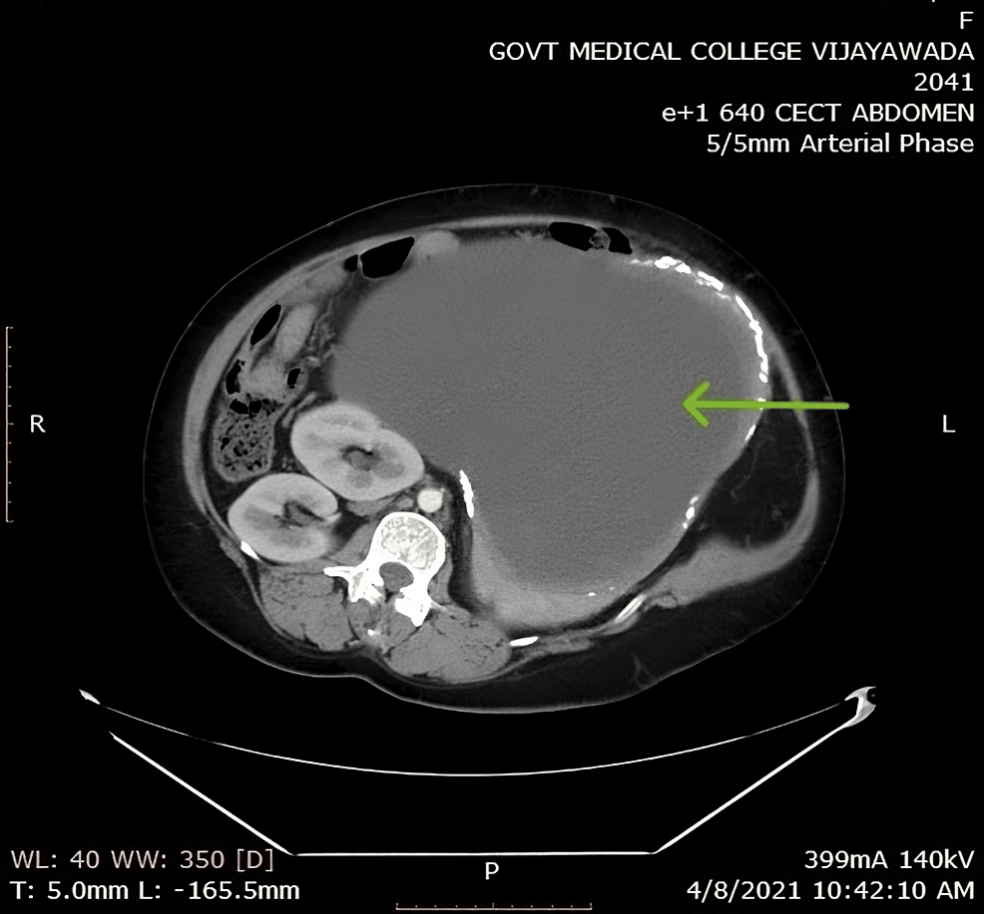
A CECT abdomen of the patients showing the splenic hydatid Axial section of a contrast-enhanced CT scan of the abdomen (green arrow pointing the splenic hydatid)

**Figure 4:**
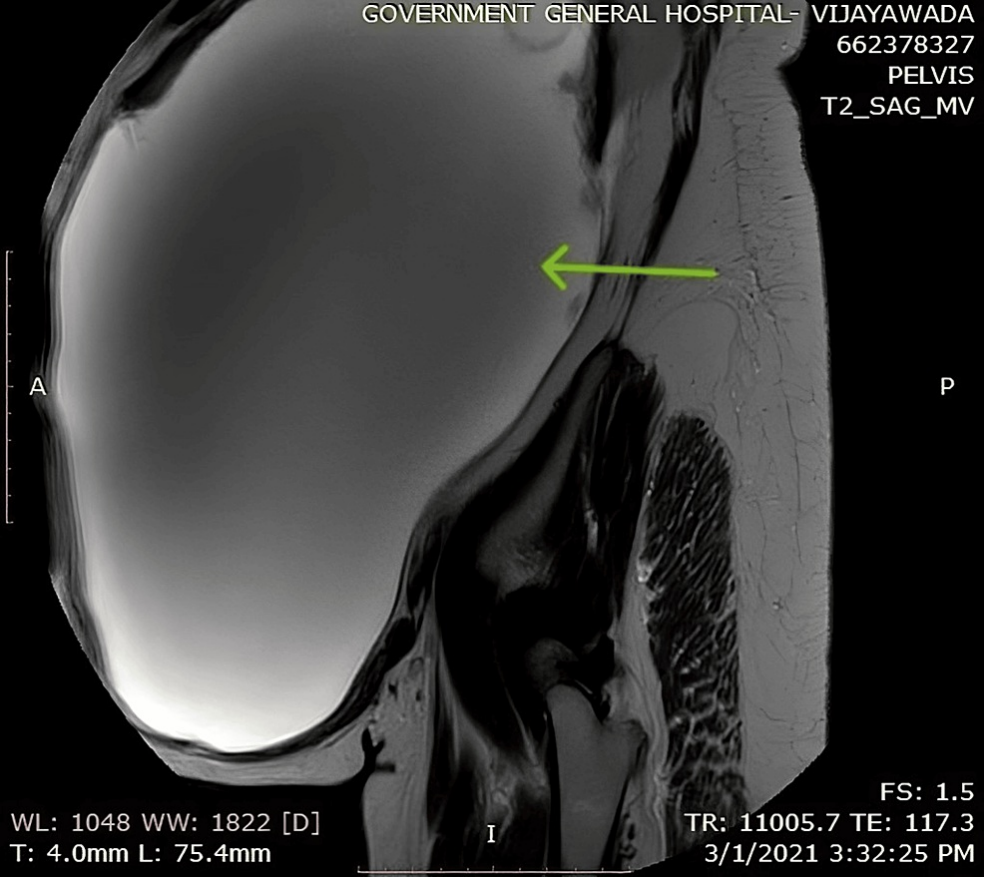
MRI abdomen and pelvis of the patient showing the splenic hydatid extending into the pelvis Sagittal section of the MRI of the abdomen (green arrow pointing at the splenic hydatid)

**Figure 5:**
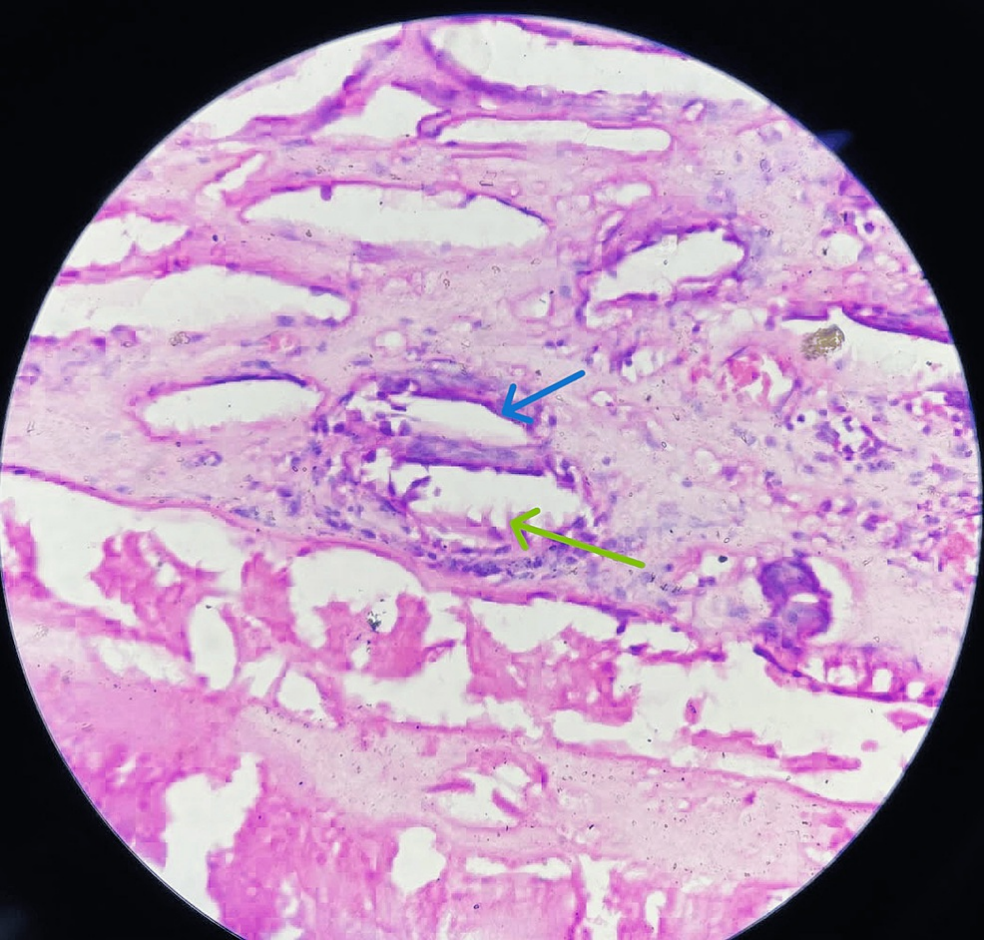
Histopathological examination image of the specimen showing capsule and hooklets Histopathological image (10x) of the specimen showing capsule (blue arrow), hooklets (green arrow)

A 21-year-old homemaker female from Andhra Pradesh, who is a meat eater was admitted to the surgical unit with complaints of pain abdomen for six months and mass per abdomen for five years with intermittent fever for six months. Dragging type of pain with no radiation. On clinical examination, a lump over the abdomen extending into the left lumbar, umbilical, and left hypogastrium with no palpable upper margin was noted. Maximum tenderness was noted in the left lumbar region. Routine blood investigations were normal. A CECT abdomen showed a large fluid-filled cystic lesion with a thick-walled focal calcification of the wall (Figure 3). The cyst was extending from the pelvis into the left side of the abdomen, displacing the left kidney to the right side, abutting the spleen, and displacing the adjacent bowel loops. The lesion was measuring approximately 51 x 30 x 21cm. The left ovary was not visualized. The uterus appears bulky and shows a heterogeneously enhancing lesion of a size measuring 7.4 x 6.9 cm protruding into the endometrial cavity. CA-125 was within normal limits, ruling out malignancy. An MRI abdomen and pelvis (Figure 4) showed a large abdominopelvic cystic lesion of size 27 x 25.4 x 14 cm with thickened walls at the superior left lateral aspect of the cyst with few areas of wall thickening and mixed intensity components suspected to be arising from the right ovary likely ovarian serous cyst-adenoma along with bulky submucosal and anterior intramural fibroid. An exploratory laparotomy was performed, and the findings were a large cyst arising from spleen crossing midline. A deroofing surgery was done to preserve the spleen with careful precautions to prevent spillage of cyst content, followed by an intra-abdominal wash with 3% NaCl, considering the cyst to be a hydatid cyst. Small cysts with internal calcification and septations were noted in the cyst. The postoperative period was uneventful. The patient was kept on oral albendazole 400 mg twice a day for 28 days. Pneumococcal and influenza vaccines were provided postoperatively. The biopsy proved to be a calcified hydatid cyst of the spleen with daughter cysts (Figure 5). On follow-up at the third and sixth months, the patient showed no recurrence or specific complaints.

Case 3

**Figure 6:**
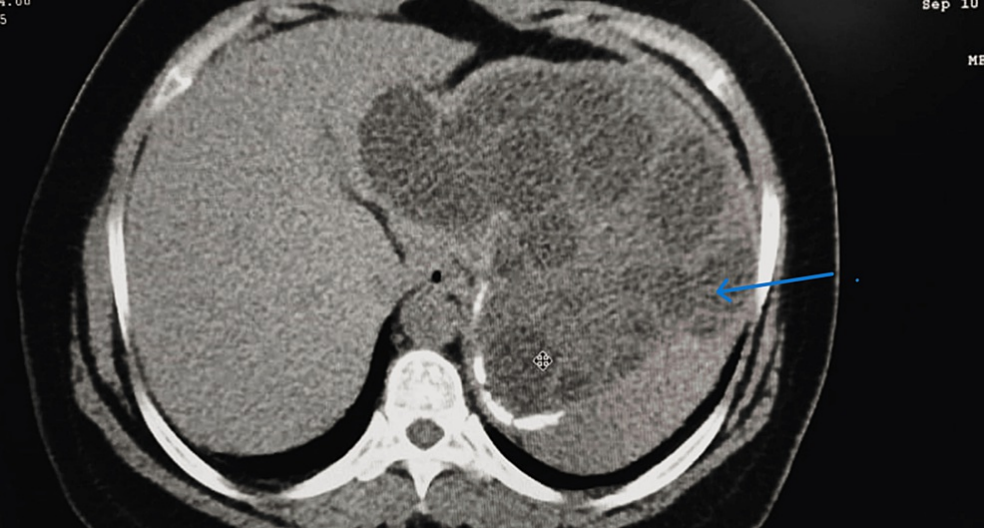
A CT scan of the abdomen showing the splenic hydatid with brood capsules (blue arrow)

**Figure 7:**
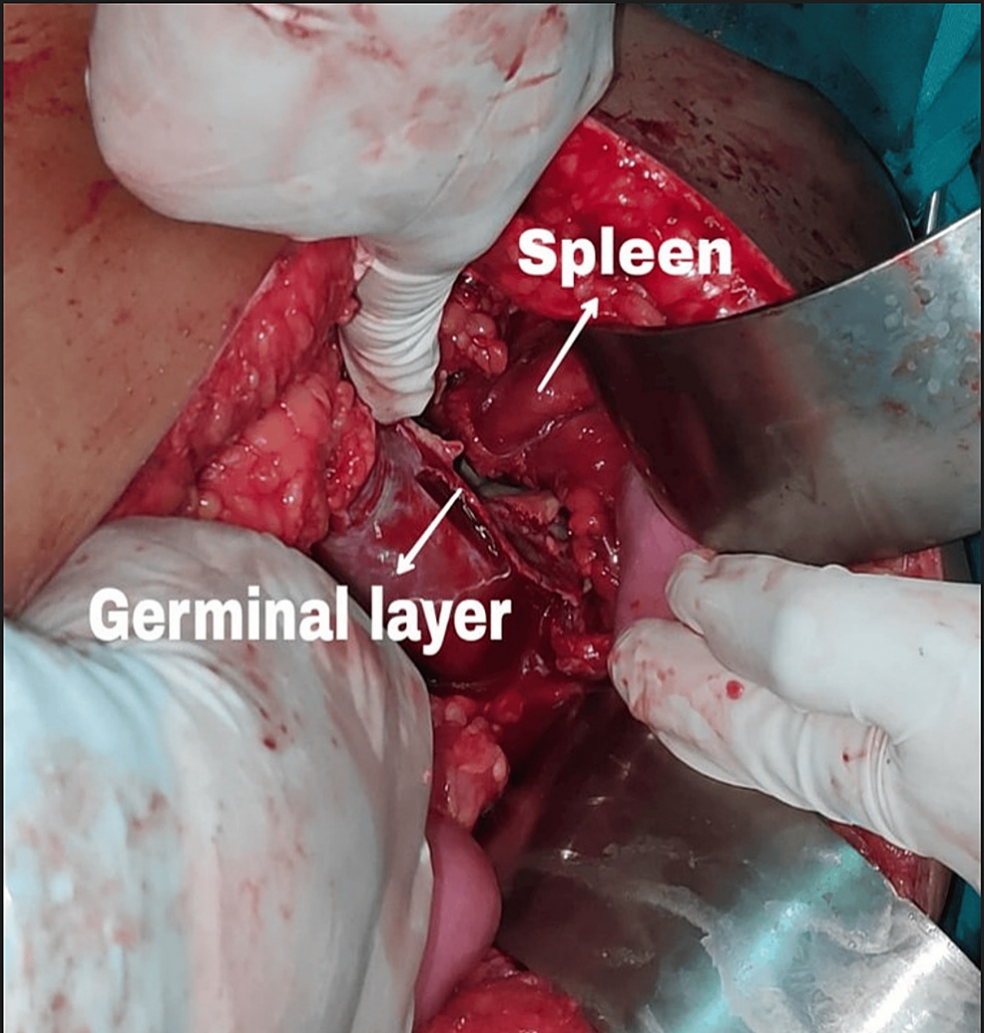
Intraoperative image showing the spleen and the hydatid capsule (germinal layer)

**Figure 8:**
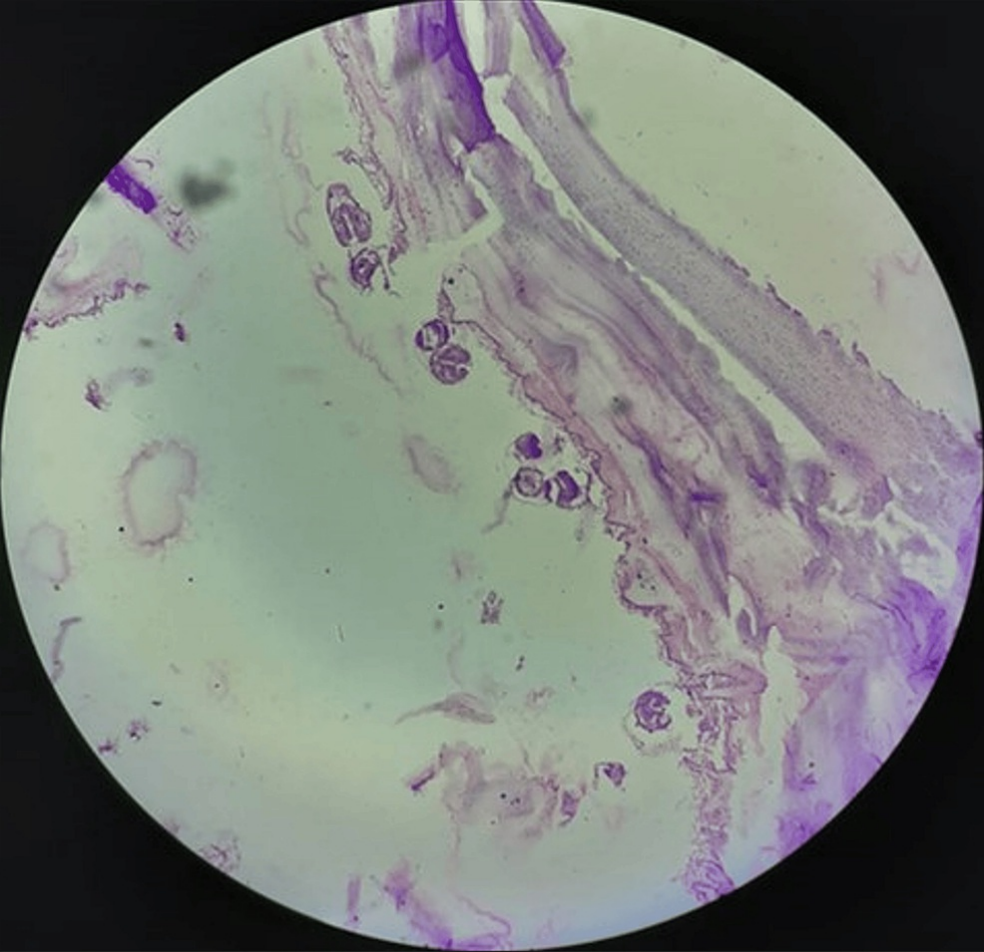
Histopathology image of the specimen

A 45-year-old female from Andhra Pradesh, who is a meat handler, presented with swelling and pain in the epigastrium and left hypochondrium for six months. She has no history of pet dogs or sheep at home. On palpation, there was mild tenderness, no hepatomegaly, and the spleen was enlarged 13 cm below the left costal margin, and a dull note was heard on percussion. Her erythrocyte sedimentation rate (ESR) was 45 mm in the first hour, her WBC count was 11,000, and the other blood investigations were normal. A CT scan of the abdomen revealed splenomegaly (13 cm) with an 11.5 x 10 cm cystic mass noted in the medial aspect of the spleen filled with multiple cysts of varied sizes with mild wall calcification and indenting the stomach, left lobe of the liver, and tail of pancreas, suggestive of splenic hydatid cyst (Figure 6). There was no evidence of the involvement of other organs. A CT scan of the chest was normal. Laparotomy was done with a vertical midline incision above the umbilicus. The cyst was visualized in the medial aspect of the spleen. After aspiration of the fluid, the cyst wall was carefully incised, and daughter cysts were removed, and complete aspiration of the fluid was done. Deroofing of the cyst with omentoplasty was done, and the abdomen was treated with hypertonic saline (Figure 7). Histopathology of the cyst revealed a laminated cyst wall encircling many scolices with a double layer of hooklets, which confirmed the diagnosis of the hydatid cyst (Figure 8). The patient was discharged and started on daily albendazole 400 mg therapy for four weeks and followed up for two years. No recurrence was found in the ultrasonogram.

## Discussion

The prevalence of hydatid disease is highest in regions where sheep grazing is prevalent on a global scale [4]. The accidental acquisition of Echinococcus granulosus by humans occurs when they inadvertently consume eggs from undercooked pigs or beef, which leads to the establishment of the parasite within their bodies. The larvae, upon hatching from their eggs, traverse the mucosal lining of the gastrointestinal tract and thereafter enter the portal system, facilitating their distribution to other organs throughout the body. Hydatid disease has the potential to impact various organs within the human body. Cysts have been observed in several organs, with the liver exhibiting the highest prevalence at 75% [5]. Other organs affected include the lungs (15.4%), muscle (5%), heart (0.5%-2%), kidney (2%), brain (1%), and spleen (1%) [5]. The prevalence of splenic involvement by hydatid cysts is significantly lower compared to other abdominal viscera. The occurrence of splenic hydatid cysts arises when the oncospheres traverse two sequential filtration barriers, namely, the liver, and lungs, and subsequently become entrapped within the splenic sinusoids. There is a suggestion that retrograde venous spread may occur from the liver to the portal vein and then through the splenic vein to the spleen. In our scenario, the growth of the cyst exerts pressure on the segmental arteries of the spleen, resulting in the infarction of its components. Furthermore, the application of force on neighboring organs such as the stomach, pancreas, kidney, and diaphragm, as well as the inflammatory response of the pericyst about these organs, can lead to the development of thick adhesions and the formation of fistulas. In this case, the presence of compression over the stomach has resulted in the occurrence of vomiting as well as compression of the IVC. One potential life-threatening complication that can arise is anaphylactic shock, which can be triggered by either spontaneous or violent rupture of the cyst [6]. Splenomegaly caused by hydatid disease is typically characterized by an absence of symptoms. The diagnosis is primarily fortuitous when doing an inquiry into other symptoms. When the cyst reaches a significant size, the individual experiences the development of a painful mass in the left hypochondrium. The initial indications of splenic hydatidosis encompass a mass in the left hypochondriac region, persistent and dragging pain, dyspepsia, heartburn, constipation, and dyspnea, as well as potential complications such as infection, rupture, or fistulization to the colon, renal artery constriction, and systemic hypertension. Additional splenic cystic lesions that should be considered include simple cysts, epidermoid cysts, abscesses, hematomas, and neoplasms. The utilization of imaging techniques, in conjunction with immunological tests, plays a crucial role in the diagnostic process of splenic hydatid. The confirmation of the diagnosis is achieved by the utilization of abdominal ultrasonography and CT scans [7].

During abdominal ultrasonography, the presence of a splenic hydatid cyst can manifest as a spherical cystic lesion with anechoic properties, accompanied by a hyperechoic border calcification. The presence of a cystic lesion, either with or without daughter cysts within the spleen, has been verified using abdominal CT imaging. The Casoni test and the enzyme-linked immunosorbent assay (ELISA) are two more assays that can be utilized in laboratory settings. When a patient presents with splenomegaly, it is imperative to examine the atypical manifestation of a solitary hydatid cyst in the spleen. In a study conducted by Balik et al. [8], the ultrasonogram showed a sensitivity of 97%, whereas the contrast-enhanced CT (CECT) of the abdomen exhibited a sensitivity of 100%. The choice of treatment is contingent upon various factors such as the stage, localization, size, and complications associated with the cysts. Chemotherapy is considered the primary treatment option for disseminated disease and for patients who possess an exceedingly high risk for surgical intervention. Albendazole, a pharmaceutical substance with antiparasitic properties, is often regarded as the preferred chemotherapy medicine. The standard recommended dosage often ranges from 10 to 15 milligrams per kilogram per day. Most antihelminthic drugs have limited absorption inside the gastrointestinal tract, resulting in inadequate concentrations within the cyst cavity to effectively eliminate the parasites [9]. Because of the limited efficacy of oral medication, the established treatment modality for hydatid cyst disease is surgical intervention, which can be performed by open or laparoscopic approaches. Splenectomy is considered the optimal therapeutic approach, and it is imperative to ensure that adequate steps are undertaken throughout the surgical procedure. The surgical care of hydatid illness encompasses several key principles, which include the complete obliteration of the cyst, the prevention of intraoperative transfer of parasites, the removal of viable membranes, and the identification of potential communication pathways. The aforementioned steps serve to inhibit the transmission of pathogens, the dispersal of contaminants, and the occurrence of anaphylactic shock. This intervention effectively mitigates the occurrence of illness and mortality during the postoperative phase. The decision between splenectomy and spleen-preserving therapy in surgical cases is determined by the specific characteristics of each case. Various surgical techniques can be employed to preserve the spleen, such as parietal splenectomy, cyst enucleation, deroofing of cyst with omentoplasty, or external drainage. Mejri et al. conducted a study advocating for the utilization of total splenectomy, particularly by the laparoscopic approach, accompanied by certain peri-operative therapeutic interventions [10].

## Conclusions

In splenic cysts occurring in endemic zones, hydatid disease should be considered as a differential diagnosis. The management of isolated hydatid cysts of the spleen should be undertaken based on the size, extent, and symptoms. Diagnosis can be challenging particularly if the hydatid involves atypical organs such as the spleen. Patients with dull aching pain on the left side of the abdomen can be suspected of splenic hydatid especially if they belong to the cattle handling occupation. A CT scan can be of great diagnostic use. Management involves both medical and surgical therapies. Antihelminthic drugs preoperatively and postoperatively should be given to prevent recurrences. Surgery is the mainstay of treatment. Laparoscopic splenectomy is the best surgical therapy for hydatid disease of the spleen. Other options include de-roofing of the cyst with or without omentoplasty, and splenectomy is the last resort.

## References

[1] Kayal A, Hussain A (2014). A comprehensive prospective clinical study of hydatid disease. ISRN Gastroenterol.

[2] Gessese AT (2020). Review on epidemiology and public health significance of hydatidosis. Vet Med Int.

[3] Baruah A, Sarma K, Barman B (2020). Clinical and laboratory presentation of hydatid disease: a study from northeast India. Cureus.

[4] Islami Parkoohi P, Jahani M (2018). Epidemiology and clinical features of hydatid cyst in northern Iran from 2005 to 2015. Iran J Parasitol.

[5] Karami M, Sadatmadani SF, Kouhi H, Sadeghi B, Rostamiyan Z, Hashemzadeh M (2021). A rare presentation of hydatid cyst, involvement of uncommon sites with sparing of typical locations. J Res Med Sci.

[6] Almulhim AM, John S (2023). Echinococcus granulosus. StatPearls.

[7] Rasheed K, Zargar SA, Telwani AA (2013). Hydatid cyst of spleen: a diagnostic challenge. N Am J Med Sci.

[8] Balik AA, Başoğlu M, Celebi F, Oren D, Polat KY, Atamanalp SS, Akçay MN (1999). Surgical treatment of hydatid disease of the liver: review of 304 cases. Arch Surg.

[9] Gomez I Gavara C, López-Andújar R, Belda Ibáñez T (2015). Review of the treatment of liver hydatid cysts. World J Gastroenterol.

[10] Mejri A, Arfaoui K, Omry A (2021). Acute intraperitoneal rupture of hydatid cysts of the liver: case series. Medicine (Baltimore).

